# Uniformity in License to Practice Medicine

**Published:** 1899-11

**Authors:** 


### Uniformity in License To Practice Medicine.

DETROIT, MICH., October 4, 1899.

*Editor Texas Medical Journal:*

DEAR SIR—The idea of uniformity of the requirements for the license to practice medicine throughout the United States is an old one. The efforts in this direction, however, seem not to have been accompanied by the desired result. After all appearances the time is come for taking further steps in this direction. It is evident that the medical profession regards the uniformity of the requirements not only as desirable, but as absolutely necessary, for several reasons.

The Wayne County (Michigan) Medical Society was so strongly impressed by the necessity of this measure, that it appointed a committee of five to investigate the question.

Circulars which have been sent to the authorities in the different States and Territories met, to a great extent, with very satisfactory preliminary replies.

The blank contained seven questions, of which 5, 6 and 7 were the most important:

5. Would you be inclined to favorably consider the plan of entering into a state of reciprocity with other States (or territories) which have practically the same requirements for the license of practicing medicine as your State (resp. territory) has?

6. Would you join in the efforts in working out a memorandum to be presented to the legislative bodies of the different States with the view of introducing a bill as to the subject matter, and would your Secretary co-operate with us?

7. Have you any sugggestions to make?

Up to September 14, answers were received from thirty-nine States and Territories. Favorable answers to question 5 or 6, 34; unfavorable answers to questions 5 and 6, 0; favorable answers to question 5, 30; favorable answers to question 6, 30; unfavorable answers to question 5, 4; unfavorable answers to question 6, 1.

The unfavorable answers were accompanied by explanations which give hope that the difficulties might be overcome which, at present, did not allow a favorable reply.

We also met with approval and encouragement from other sources. The Wayne County Medical Society takes the liberty to suggest that the medical press advocate the matter and systematically pay attention to the details which might present themselves. We further suggest that the matter be taken up by all medical societies in the country, and as we are unable to reach all of them, we ask for your assistance.

The Wayne County Medical Society solicits your coöperation and suggestions, for which kindly accept our thanks in advance.

Very respectfully yours,

E. AMBERG, M. D.,

32 West Adams Ave. Secretary of Committee.

REPORT OF COMMITTEE, SEPTEMBER 14, 1899.—BY DR. AMBERG, SECRETARY OF THE COMMITTEE.

The committee appointed by the Wayne County Medical Society met several times. It appeared that the time was too short to follow up the two questions put by the society, therefore only the Uniformity question was taken up. The committee sent to the authorities in the fifty-one States and Territories circular letters and blanks.

The committee is glad to acknowledge the prompt answers which have been received.

Naturally the answers could only be of a preliminary character.

The committee begs to submit to the Society the suggestion to express their thanks to the parties concerned, and to send them the report of the committee.

Answers have been received from thirty-six States and three Territories.

Favorable answers to questions 5 or 6, 34; unfavorable answers to questions 5 or 6, 0; favorable answers to question 5, 30; unfavorable answers to question 5, 4—Arkansas, California, Colorado and Connecticut; no answers to question 5, 1—Ohio; not prepared to answer question 5, 4—Alabama, Delaware, Massachusetts and West Virginia; favorable answers to question 6, 30; unfavorable answers to question 6, 1—Kentucky; no answers to question 6, 2—New Jersey and Pennsylvania; not prepared to answer question 6, 6—Alabama, Arkansas, Delaware, Massachusetts, Mississippi and West Virginia.

It must be remarked that the unfavorable answers are accompanied by explanations which make it not at all impossible to overcome the difficulties, which, at present, do not allow a favorable reply. It must be further considered, that in some States there might be no authority in this matter, viz.; in Michigan at present. Naturally no answer could be expected from such a State. Some of the answers to question 7 might be reported in extenso. (See answers.)

After all it is the desire of, we might say, the majority of the authorities all over the United States to have the matter settled. Editorials have been noticed in the *New York Medical Journal* and in the *Physician and Surgeon.* Other parties also encouraged the movement. The committee found that there existed a National Confederation of State Medical Examining and Licensing Boards. However, the committee is of the opinion that the medical societies of the United States ought to act separately, at least for the first time, because in this way, the aim of the National Confederation can only be furthered.

Only one single State gave as a reason for not answering their membership to above mentioned society.

It was understood by the committee that the society did not expect any final results, because these can only be reached in the course of years. The movement has only been started, and we can report that the outlook is promising.

We see that many parties are now interested in the matter.

There exists also a strong desire for a better medical education. In order to bring the movement to the knowledge of wider circles in the medical profession, the committee suggests that the Wayne County Medical Society, without delay, send a circular to all the medical papers and all National and State Medical Societies in the United States, also to some of the other larger ones, reading like this: (Circular omitted.)

It appears that the laity does not quite understand that questions of this kind are of not less importance for them than they are for the medical profession. Here the press must do its part. The committee appreciates the interest the daily press is taking in the matter. We are convinced that the press can only approve of the movement and that it is aware of the fact, that in this case, not much can be accomplished without the vigorous assistance of the same. We hope the press will follow up the movement with tireless energy, because it is in the interest of the national welfare.

Geo. G. Gordon, M. D., Frank D. Summers, M D.,
E. H. Troy, M. D., E. B. Smith, M. D.,
E. Amberg, M. D.,
Committee.

